# PI3K/mTOR Dual Inhibitor PF-04691502 Is a Schedule-Dependent Radiosensitizer for Gastroenteropancreatic Neuroendocrine Tumors

**DOI:** 10.3390/cells10051261

**Published:** 2021-05-20

**Authors:** Zeta Chow, Jeremy Johnson, Aman Chauhan, Tadahide Izumi, Michael Cavnar, Heidi Weiss, Courtney M. Townsend, Lowell Anthony, Carrigan Wasilchenko, Matthew L. Melton, Jörg Schrader, B. Mark Evers, Piotr Rychahou

**Affiliations:** 1 Markey Cancer Center, University of Kentucky, Lexington, KY 40536, USA; zeta.chow@uky.edu (Z.C.); jeremy-johnson@uky.edu (J.J.); amanchauhan@uky.edu (A.C.); t.izumi@uky.edu (T.I.); Michael.Cavnar@uky.edu (M.C.); Heidi.weiss@uky.edu (H.W.); lowell.anthony@uky.edu (L.A.); carrigan.wasilchenko@uky.edu (C.W.); Matthew.Melton@uky.edu (M.L.M.); mark.evers@uky.edu (B.M.E.); 2 Department of Surgery, University of Kentucky, Lexington, KY 40536, USA; 3 Department of Internal Medicine, Division of Medical Oncology, University of Kentucky, Lexington, KY 40536, USA; 4 Department of Toxicology and Cancer Biology, University of Kentucky, Lexington, KY 40536, USA; 5 Department of Internal Medicine, Division of Cancer Biostatistics, University of Kentucky, Lexington, KY 40536, USA; 6 Department of Surgery, University of Texas Medical Branch, Galveston, TX 77555, USA; ctownsen@utmb.edu; 7 Medical Department and Department of General, Visceral and Thoracic Surgery, University Medical Center Hamburg-Eppendorf, 20251 Hamburg, Germany; jschrader@uke.de

**Keywords:** neuroendocrine tumor, radiosensitization, PI3K inhibitor

## Abstract

Patients with advanced-stage gastroenteropancreatic neuroendocrine tumors (GEP-NETs) have a poor overall prognosis despite chemotherapy and radiotherapy (e.g., peptide receptor radionuclide therapy (PRRT)). Better treatment options are needed to improve disease regression and patient survival. The purpose of this study was to examine a new treatment strategy by combining PI3K/mTOR dual inhibition and radiotherapy. First, we assessed the efficacy of two PI3K/mTOR dual inhibitors, PF-04691502 and PKI-402, to inhibit pAkt and increase apoptosis in NET cell lines (BON and QGP-1) and patient-derived tumor spheroids as single agents or combined with radiotherapy (XRT). Treatment with PF-04691502 decreased pAkt (Ser473) expression for up to 72 h compared with the control; in contrast, decreased pAkt expression was noted for less than 24 h with PKI-402. Simultaneous treatment with PF-04691502 and XRT did not induce apoptosis in NET cells; however, the addition of PF-04691502 48 h after XRT significantly increased apoptosis compared to PF-04691502 or XRT treatment alone. Our results demonstrate that schedule-dependent administration of a PI3K/mTOR inhibitor, combined with XRT, can enhance cytotoxicity by promoting the radiosensitivity of NET cells. Moreover, our findings suggest that radiotherapy, in combination with timed PI3K/mTOR inhibition, may be a promising therapeutic regimen for patients with GEP-NET.

## 1. Introduction

Gastroenteropancreatic neuroendocrine tumors (GEP-NETs) are heterogeneous clinical and pathological subsets of NETs arising from the gastrointestinal tract. Historically, NETs have been considered an “orphan” disease [1]. However, the incidence of these tumors increased approximately 6.4-fold from 1973 to 2012, which is likely attributed to advanced imaging and earlier diagnosis [2]. Among all NETs, GEP-NETs have the highest incidence, at 3.56 per 100,000 population [2]. In addition, many patients experience diagnostic delays due to the lack of specific presenting symptoms. In contrast to these early-stage asymptomatic patients, symptomatic patients are often diagnosed with advanced or metastatic disease and have a poor overall prognosis [3].

Peptide receptor radionuclide therapy (PRRT) has emerged as an effective therapeutic modality for treating GEP-NETs, with clinical trials showing increased progression-free survival and overall survival in patients [4,5,6,7,8]. Somatostatin receptors are highly expressed in 80–100% of GEP-NETs [9,10]. Synthetic somatostatin analogues, which have a high affinity to GEP-NETs, and coupled with a radioactive element, facilitate uptake into cells to deliver targeted intracellular radiation [7,9]. The PRRT compounds currently available in the United States and approved for treatment of GEP-NETs include ^177^Lu-Octreotide, ^90^Y-DOTATOC, and ^111^In-DTPA-octreotide [9]. ^177^Lu-Octreotide has achieved partial and minor response rates of 29% and 16%, respectively, and improved progression-free survival and overall survival in the NETTER-1 phase III clinical trial, thus indicating the utility of PRRT as a potential treatment option for inoperable or metastatic disease [8,9]. Another evolving approach in cancer treatment is to utilize a multimodal regimen to enhance the effects of single agents for tumor regression. For example, radiosensitizers, such as 5-FU, capecitabine, and temozolomide, have been tested in conjunction with PRRT [11,12,13].

The mammalian target of rapamycin (mTOR) and the PI3K/mTOR/Akt signaling pathway play a role in tumor proliferation, survival and angiogenesis [14]. Treatment with everolimus, a potent inhibitor of the mTORC1 subunit, significantly improved progression-free survival in the RAD001 in advanced neuroendocrine tumors (RADIANT) clinical trials [15,16,17,18,19,20]. Of particular interest, mTOR inhibitors have been tested in vitro as radiosensitizers in combination with PRRT [21]. Recently, PRRT combination therapy with everolimus has entered phase I/II clinical trials [22,23]. Although this combination therapy has demonstrated an improved treatment response rate of 44%, serious side effects, such as neutropenia and renal function impairment, occurred in 8 out of 11 patients in one study, requiring dose reduction [22,23]. New combination strategies may improve patient tolerance to PI3K/mTOR, such as inhibiting PI3K/mTOR pathway only when cancer cells experience radiation-induced stress, which may offer short-term synergistic effects combined with radiotherapy.

Novel and promising PI3K and mTOR dual inhibitors with advanced antineoplastic properties, such as PF-04691502 and PKI-402, inhibit multiple targets in the PI3K/mTOR/Akt pathway and may be superior therapeutic agents compared to everolimus [24]. PI3K/mTOR dual inhibitors have been assessed in several clinical trials as a treatment for various advanced cancers, but rarely GEP-NET [25,26,27,28]. Furthermore, little is known about the effect of PI3K/mTOR inhibition and radiosensitization of GEP-NET cells. In this study, we evaluated the response of GEP-NET cells to the combination of a novel PI3K/mTOR inhibitor in conjunction with radiotherapy. Our results show that schedule-dependent administration of a dual PI3K/mTOR inhibitor and radiotherapy can improve treatment response by increasing apoptosis in vitro. Furthermore, our results suggest that this combination strategy may be a potential treatment to improve disease regression and progression-free survival among GEP-NET patients.

## 2. Materials and Methods

### 2.1. Materials and Reagents

Tissue plates were from Olympus (Olympus Cooperation, Tokyo, Japan): 6-well flat bottom tissue culture plate (25–105), 96-well flat bottom tissue culture plate (25–109); Corning (Corning Inc, New York, NY, USA): 24-well low attachment plates (CLS3473), 96-well clear round bottom ultra-low attachment microplate (7007) and Millipore Sigma (MilliporeSigma, Burlington, MA, USA): collagen IV-coated plates (collagen from human placenta; C7521). A sterile cell strainer 70 µm nylon mesh was from FisherBrand (Fisher Scientific, Hampton, NH, USA; 22-363-548). The cell culture medium and reagents included: Roswell Park Memorial Institute (RPMI) 1640 medium (Gibco Thermo Fisher Scientific, Waltham, MA; 11875085), DMEM/F12-GlutaMAXTM medium (Gibco Thermo Fisher Scientific, Waltham, MA, USA; 10565018), HyClone™ HEPES (GE Healthcare, Chicago, IL, USA; SH30237.01), IntestiCult organoid growth medium (Stemcell Technologies, Vancouver, Canada; 06010), recombinant human FGF-basic (Peprotech, Rocky Hill, NJ, USA; 100-18B), epidermal growth factor (BioVision, Milpitas, CA, USA; 4022), non-essential amino acid solution (Millipore Sigma, Burlington, MA, USA; M7145), sodium pyruvate solution (Millipore Sigma, Burlington, MA, USA; S8636), MEM vitamin solution (Millipore Sigma, Burlington, MA, USA; M6895), Liberase DH (Roche Applied Science, Penzberg, Germany; 05401054001), collagenase/hyaluronidase (StemCell Technologies, Vancouver, Canada; 07912), trypsin-EDTA solution (Sigma-Aldrich, St. Louis, MO, USA; SLCB7154), antibiotic-antimycotic 100 X (Gibco Thermo Fisher Scientific, Waltham, MA, USA; 15240-062), fetal bovine serum (FBS) (Sigma-Aldrich, St. Louis, MO, USA; 12303C-500), Accumax (Innovative Cell Technologies, San Diego, CA, USA; AM105).

PI3K/mTOR dual inhibitors used in this study were PF-04691502 (Selleckchem, Houston, TX, USA; S2743) and PKI-402 (Selleckchem, Houston, TX, USA; S2739). Drugs were diluted in dimethyl sulfoxide (DMSO) (Fisher Scientific, Waltham, MA, USA; D128-500) and stored at −80 °C prior to use. A cell medium mixture with DMSO only was added to cells in the control group.

Western blot materials included: Dulbecco’s phosphate buffered saline (PBS) (Sigma-Aldrich, St, Louis, MO, USA; D8637-500), RIPA buffer 10X (Cell Signaling Technology, Danvers, MA, USA; 9806S), protein assay dye (Bio-Rad Laboratories, Hercules, CA, USA; 5000006), MOPS SDS running buffer 20 X (Invitrogen, Carlsbad, CA, USA; NP0001-02), Tris-Glycine 10 X transfer buffer solution (Fisher Scientific, Waltham, MA, USA; BP1306-1), TBS buffer 20 X (VWR International, Radnor, PA, USA; J640-4L), Tween 20 (Fisher Scientific, Waltham, MA, USA; BP337-500), NuPAGETM 4–12% Bis-Tris Gel 1.0 mm × 10 well (Invitrogen, Carlsbad, CA, USA; NP0321B0X), NuPAGETM LDS sample buffer 4 X (Invitrogen, Carlsbad, CA, USA; NP0007), NuPAGETM sample reducing agent (Novex Products Inc, Lorain, OH, USA; NP0009), phenylmethanesulfonyl fluoride (PMSF) solution 100 X (Sigma-Aldrich, St. Louis, MO, USA; 93482-50), Precision Plus ProteinTM dual color standards (Bio-Rad Laboratories, Hercules, CA, USA; 161-0374), sodium azide (Fisher Scientific, Waltham, MA, USA; S2271-100), non-fat dry milk (Lab Scientific, Danvers, MA, USA; M0841), methanol (VWR International, Radnor, PA, USA; BDH1135-4LP), Amersham ECL prime Western blotting detection reagent (GE Healthcare Life Sciences, Chicago, IL, USA; RPN2209), and Immobolin Western (Millipore Sigma, Burlington, MA, USA; WBKLS0500). The following primary antibodies were from 1) Abcam (Cambridge, United Kingdom): anti-chromogranin A (ab45179, 1:500), recombinant anti-cyclin D1 antibody (ab134175, 1:5000); 2) Santa Cruz Biotechnology Inc (Dallas, TX, USA): SSTR2 antibody (A-8) (SC-365502, 1:100), SYP antibody (4H255) (SC-58301, 1:100); and 3) Cell Signaling Technology (Danvers, MA, USA): phospho-Akt (Ser473) (D9E) XP ^®^ rabbit mAb (4060, 1:1000), Akt (pan) (40D4) mouse mAb (2920, 1:1000), S6 ribosomal protein (5G10) rabbit mAb (2217, 1:1000), 4E-BP1 (53H11) rabbit mAb (9644, 1:1000), phospho-4E-BP1 (Thr37/46) (236B4) rabbit mAb (2855, 1:1000), phospho-S6 ribosomal protein (Ser235/236) (D57.2.2E) XP ^®^ rabbit mAb (4858, 1:2000), cleaved PARP (Asp 214) (D64E10) XP^®^ rabbit mAb (5625, 1:1000), PARP (46D11) rabbit mAb (9532, 1:1000), β-Actin (8H10D10) mouse mAb (3700, 1:5000) and GAPDH (14C10) rabbit mAb (2118, 1:1000). Secondary antibodies were from Santa Cruz Biotechnology (Dallas, TX, USA): mouse anti-rabbit IgG-HRP (SC-2357), and m-IgGk BP-HRP (SC-516102).

Proliferation and colorimetric cell survival analyses were performed with CytoScan**^TM^** SRB Cell Cytotoxicity Assay (G-Biosciences, Geno Technology Inc, St. Louis, MO, USA; 786-213) [29].

CCK-8 viability analysis was performed with CCK-8 reagent (MedChemExpress LLC, Monmouth Junction, NJ, USA; HY-K0301).

A DNA fragmentation ELISA photometric enzyme immunoassay was performed with the Cell Death Detection ELISA^PLUS^ kit (Roche Holding AG, Basel, Switzerland; C755B93).

Cell count and viability were determined by the Vi-Cell BLU cell viability analyzer (Beckman Coulter Life Sciences, Brea, USA). Photospectrometry was conducted by the Varioskan LUX microplate reader (ThermoFisher Scientific, Waltham, MA, USA).

### 2.2. Cell Culture

The BON cell line was derived from a human pancreatic neuroendocrine tumor and previously characterized [30,31]. QGP-1, a pancreatic neuroendocrine cell line purchased from Japan Health Sciences Foundation [32], was maintained in ATCC-formulated RPMI 1640 medium with 10% fetal bovine serum and 1% 100 × penicillin antibody solution. The NT-3 cells, derived from a human pancreatic NET, were a kind gift from Dr. Jörg Schrader (University Medical Center Hamburg-Eppendorf, Hamburg, Germany). All cells were cultured in a humidified incubator at 37 °C and 5% CO_2_. QGP-1 cells were cultured in RPMI-1640 + 10% FBS + 1% 100 × penicillin antibody solution. BON cells were cultured in a 1:1 mixture of DMEM F1/2 GlutaMAX supplemented with 10% fetal bovine serum and 1% 100 × penicillin antibody solution in 5% CO_2_ at 37 °C. NT-3 cells were cultured in RPMI medium supplemented with 10% FBS, 10 mM HyClone™ HEPES, 1X antibiotic-antimycotic, 20 ng/mL epidermal growth factor, 10 ng/mL recombinant human FGF-basic. NT-3 cells were cultured on collagen IV-coated plates. Collagen IV solution was prepared in PBS for coating cell culture plates at 50 µg/mL concentration [33].

### 2.3. NET Tumor Spheroids

Patient samples were collected under Markey Cancer Center Tissue Collection for Tumor Research protocol (IRB #44231). The original patient NET tumor (F0 generation) was divided into 2 mm^3^ pieces and digested in 50 µg/mL Liberase DH (100 µL) and 0.5 X collagenase/hyaluronidase (250 µL), diluted in 5 mL of DMEM/F12 serum free media for 4 h at 37 °C with gentle agitation by a magnetic stirring bar. Liberase DH was resuspended in sterile water (2.5 mg/mL concentration) and stored in single-use 100 µL aliquots at −80 °C. Collagenase/hyaluronidase was aliquoted into single-use 250 µl aliquots and stored at −80 °C. Digested cells were washed twice with complete cell culture media and transferred into 24-well low-attachment plates in 10% DMEM/F12 FBS, 1 X MEM non-essential amino acid solution, 10 mM sodium pyruvate solution, 1 X MEM vitamin solution, 10 mM HyClone™ HEPES. Cell culture media were supplemented with 1 X antibiotic-antimycotic, 20 ng/mL epidermal growth factor, 10 ng/mL recombinant human FGF-basic. IntestiCult organoid growth medium was added to each well of the 24-well plates in a 1 to 4 proportion to culture media.

### 2.4. Radiotherapy

Cells were irradiated using a Precision X-Ray irradiator (X-RAD-225XL, North Branford, CT, USA) at the X-ray Service Center of the Department of Toxicology and Cancer Biology of the University of Kentucky (Ref: 32438621 and 30673636). The energy of the X-ray used was 225 kV at a dose rate of approximately 1.7 Gy/min. Accurate absorbed doses were calculated by considering the impact of backscattering as previously described [34]. To determine radiation dose-dependent apoptosis, cells were irradiated (2, 4, and 8 Gy) once, followed by incubation for 48, 72, and 96 h. To assess the synergistic effect of radiotherapy and PF-04691502, cells were irradiated (2 Gy) once, incubated for 48, 72, and 96 h, followed by PF-04691502 treatment (500 or 1000 nM) for 24 h. To determine DNA fragmentation, a marker for induced cell death and apoptosis, cells were irradiated (2 Gy), incubated for 96 h, followed by 500 nM PF-04691502 treatment for 24 h. Finally, to determine the synergistic effect of simultaneous radiotherapy and PF-04691502, cells were irradiated (2 and 4 Gy) and immediately treated with PF-04691502 (500 nM or 1000 nM), followed by incubation for 24 h.

### 2.5. Immunohistochemistry

GEP-NET patient tissue samples (*n* = 39) were identified by the Markey Cancer Center Biospecimen Procurement and Translational Pathology Shared Resource Facility. Immunohistochemistry (IHC) was performed as previously described [35]. Briefly, slides were deparaffinized in xylene, rehydrated, incubated for 15 min with fresh 0.3% hydrogen peroxide, washed with PBS, and heated to 95 °C for 30 min in sodium citrate buffer (10 mM sodium citrate, 0.05% Tween 20, pH 6.0).

Endogenous peroxidase activity was blocked with Bloxall blocking solution (Vector Laboratories, Burlingame, CA, USA; SP-6000). Next, sections were blocked for 1 h with 2.5% normal horse serum (Vector Laboratories, Burlingame, CA, USA; S-2012). pAkt (Ser473) antibody was diluted in Dako background reducing antibody diluent (Agilent Dako Products, Santa Clara, CA, USA; S3022). Primary antibody was incubated with slides for 12 h at 4 °C in a humidifier chamber, washed with Tris-buffered saline and Tween 20 (TBST) and incubated with ImmPRESS universal antibody IgG polymer detection kit (Vector Laboratories, Burlingame, CA, USA; MP-7500) for 1 h at room temperature. Antibody reaction was visualized with Immpact DAB EqV peroxidase substrate (Vector Laboratories, Burlingame, CA, USA; SK-4103). All sections were counterstained with hematoxylin (VWR international, Radnor, PA, USA; 95057-844) and observed by light microscopy. For negative controls, primary antibody was omitted from the above protocol.

The number of positive cells was visually evaluated in each core by a pathologist, and the staining intensity was classified using a semi-quantitative seven-tier system developed by Allred et al. [36,37]. The system assesses the percentage of positive cells (none = 0; <10% = 1; 10% to 50%, = 2; >50% = 3) and intensity of staining (none = 0; weak = 1; intermediate = 2; and strong = 3).

### 2.6. Immunoblotting

Cells were seeded at 800,000/2 mL in 6-well plates. To determine inhibition of the mTOR/pAkt pathway, cells were treated with either PKI-402 (50 to 1000 nM) or PF-04691502 (100 to 10000 nM) for 24, 48, and 72 h and selected tumor spheroids were treated with PF-04691502 (500 nM) for 24 h prior to lysis. To examine markers for NET origin, the selected tumor spheroids were incubated in a humidified 37 °C 5% CO_2_ incubator for 7 d prior to lysis. To determine the synergistic effect on apoptosis, cells were treated by means of radiotherapy or radiotherapy combined with PF-04691502 as described above, prior to lysis. The immunoblotting for individual experiments was performed as previously described [38].

### 2.7. Sulforhodamine B (SRB) proliferation assay

Cells were seeded at 5000 cells/100 µL in 96-well plates and treated with PF-04691502 (100 nM to 10,000 nM), followed by incubation in a humidified 37 °C 5% CO_2_ incubator. Cells were then fixed, stained, and quantified following the Cytoscan**^TM^** SRB Cell Cytotoxicity Assay protocol [29].

### 2.8. Colorimetric cell survival assay

Cells were seeded at 500 cells/100 µL in 96-well plates and treated with PF-04691502 (25 nM to 500 nM), followed by incubation in a humidified 37 °C 5% CO_2_ incubator for 14 d. Cells were then fixed and stained followed by quantification per Cytoscan SRB cell cytotoxicity assay protocol [29].

### 2.9. CCK-8 Viability Assay

Tumor spheroids were established as described and cultured in 24-well ultra-low attachment plates. First, tumor spheroids were collected into 50 mL conical tubes and centrifuged at 2000 rpm; cell medium was removed, and tumor spheroids were resuspended in Accumax solution for 15 min at 37 °C. Tumor spheroids were gently and thoroughly pipetted with a 1 mL pipette to form a homogenous single cell suspension. Accumax solution was then neutralized with complete cell medium. The cell suspension was centrifuged at 2000 rpm, the supernatant was removed entirely, and the cells were resuspended in complete medium. Cells were passed through a sterile cell strainer 70 µm nylon mesh to remove any undigested tumor spheroids. Cells were seeded at 10,000 cells/100 µL density per well into a 96-well clear round bottom ultra-low attachment microplate to allow the formation of spheroids over the next 24 h. Finally, tumor spheroids were treated with 1000 nM PF-04691502 for 48 h, followed by the addition of CCK-8 reagent (20 µL), and incubated for 72 h prior to analysis. Colorimetric change induced by CCK-8 reagent, which is directly proportional to the rate of cell proliferation, was measured by absorbance at 450 nm.

### 2.10. DNA Fragmentation ELISA Photometric Enzyme Immunoassay

Cells were seeded at a 1000 cells/100 µL density in 96-well plates and treated by radiotherapy alone or radiotherapy combined with PF-04691502 as described above prior to quantification, as per the ROCHE Cell Death Detection ELISA^PLUS^ protocol [39].

### 2.11. Statistical Analysis

Descriptive statistics, including means and SD, are presented in each experimental group and displayed in bar graphs. Percentage inhibition was calculated compared to the control of each experiment and plotted against concentrations in a logarithmic scale. The standard curves and absolute maximal inhibitory concentration (IC-50) values were generated using SigmaPlot software version 14 (Systat Software Inc., San Jose, CA, USA). Immunohistochemistry scores were summarized descriptively. Comparisons of SRB absorbance, proliferation, colorimetric survival assay, CCK-8 viability assay, and DNA fragmentation ELISA enzyme immunoassay were performed using one and two-way analysis of variance (ANOVA) with Holm’s adjustment for multiple testing between groups. *P* < 0.05 was considered to indicate a statistically significant difference. Statistical analyses were performed using SAS software version 9.4 (SAS Inc., Cary, NC, USA).

## 3. Results

### 3.1. PAkt (Ser473) Expression in GEP-NET Tumors

**Figure 1:**
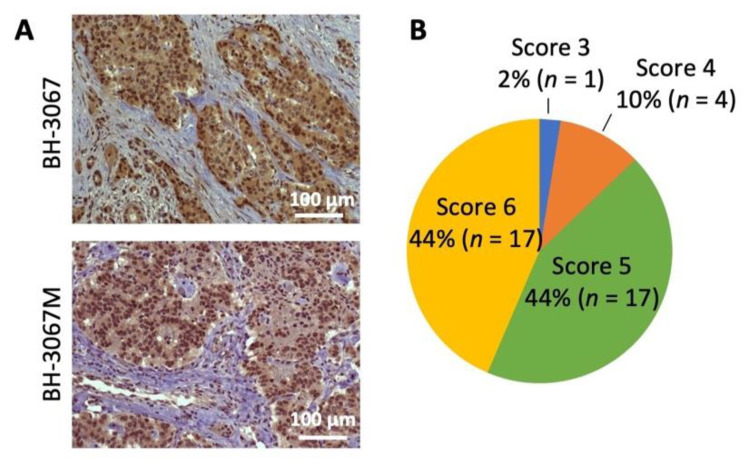
Analysis of pAkt (Ser473) expression in NET patient samples. (**A**) Representative pAkt (Ser473) immunohistochemistry staining of patient neuroendocrine tumor samples. Positive staining was observed in the cytoplasm. Patient GEP-NET samples’ (*n* = 39) scores were evaluated by a pathologist and the staining intensity was classified using a semi-quantitative seven-tier system. (**B**) Percentage distributions of the cytoplasmic expression of pAkt (Ser473) scored by abundance and intensity is shown in the pie chart.

The PI3K/Akt/mTOR pathway has been implicated in GEP-NET development and the improvement of progression-free survival [40,41]. pAkt expression was analyzed in 39 GEP-NET tumor samples; 88% of the samples were stained as strongly positive (score 5 or 6). The staining intensity was weak in 2% (score 3 or less) and intermediate in 10% of cases (score 4) (Figure 1A,B and Figure S1). Positive cytoplasmic staining was observed in all patient samples with positive pAkt staining. Although the sample number was somewhat limited, these results suggest increased Akt activation in the majority of GEP-NETs.

### 3.2. Cellular Profiling of PI3K/mTOR Inhibitors, PF-04691502 and PKI-402, in GEP-NET Cancer Cell Lines

**Figure 2:**
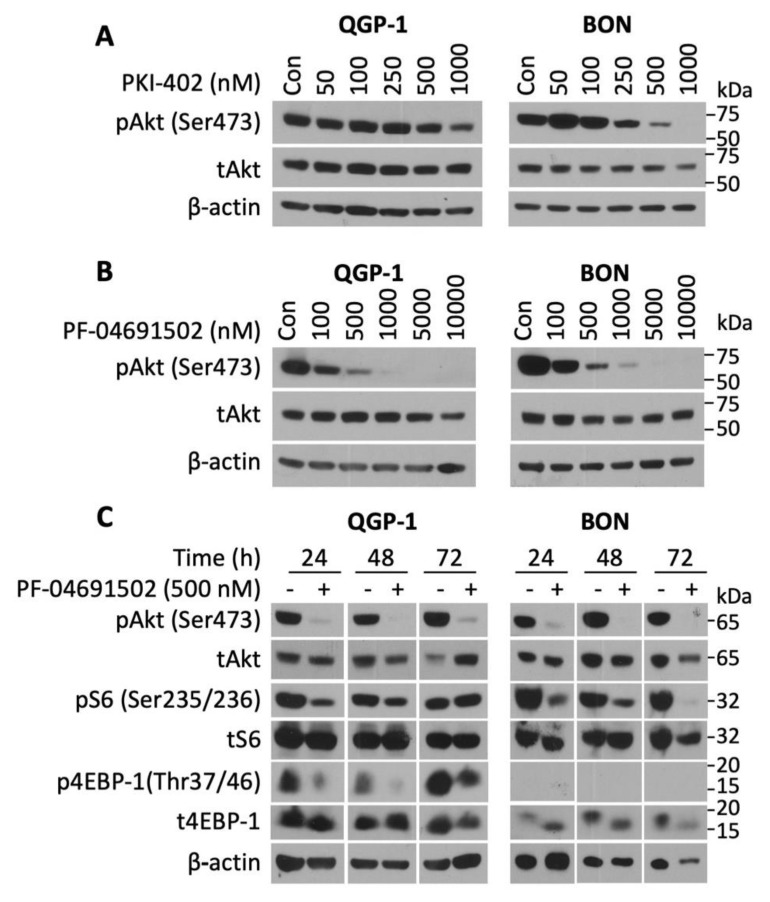
pAkt (Ser473) inhibition by PKI-402 and PF-04691502 in NET cell lines. (**A**) QGP-1 and BON cells were seeded in a 6-well plate at 800,000 cells/2 mL density and treated with PKI-402 from 50 to 1000 nM, and collected at 24 h. Western blot analysis was performed to confirm PI3K/Akt pathway inhibition at 24 h. (**B**) QGP-1 and BON cells were seeded in 6-well plates at 800,000 cells/2 mL density and treated with PF-04691502 from 100 to 10,000 nM, and collected at 24 h. Western blot analysis was performed to confirm PI3K/Akt pathway inhibition. (**C**) QGP-1 and BON cells were seeded in 6-well plates at 800,000 cells/2 mL density and treated with 500 nM PF-04691502. Protein was collected at 24, 48, and 72 h. In addition to pAkt (Ser473) inhibition, downstream targets of the PI3K/Akt pathway, pS6 (Ser235/236) and p4EBP-1 (Thr37/46) inhibition, were confirmed with Western blot analysis. β-actin was used as a loading control.

To determine the activity of the novel PI3K/mTOR inhibitors on NETs, three well-established human GEP-NET cell lines (QGP-1, BON, and NT-3) were treated with various concentrations (50–1000 nM) of either PKI-402 or PF-04691502 over a defined time period. PKI-402 (500 and 10,000 nM) completely inhibited pAkt expression at 4 h in both QGP and BON cells (Figure S2). However, this effect was not prolonged, as noted by the attenuation of pAkt expression at 24 h with only the highest concentration (i.e., 1000 nM in QGP-1 cells and 500 nM in BON cells) (Figure 2A). In contrast, PF-04691502 potently inhibited pAkt expression in both QGP-1 (at concentrations of 100 to 10,000 nM) and BON (at concentrations of 100 to 10,000 nM) cell lines at 24 h after treatment (Figure 2B). Though both PKI-402 and PF-04691502 are ATP-competitive, reversible and specific PI3K/mTOR dual inhibitors, they differ in their pharmacokinetics [24,42]. For instance, PKI-402 particularly suppresses pAkt at Thr308 and requires a higher dose to inhibit pAkt at Ser428 [42]. Since complete activation of Akt kinases occurs when the mTORC2 complex phosphorylates Akt at Ser428, our study utilized Akt inhibition at Ser428 as the marker for Akt inhibition [42].

Next, we treated QGP-1 and BON cells with PF-04691502 (500 nM) to test the duration of PI3K pathway inhibition (Figure 2C). The expression of pAkt was inhibited in both QGP-1 and BON cells at 24, 48 and 72 h. Similarly, expression of pS6 (Ser235/236), which is a key regulator of 40 S ribosome subunit biogenesis, was inhibited in both cell lines. Finally, we assessed the expression of p4EBP-1 (Thr37/46), which plays a critical role in translational mRNA complex assembly and found that PF-04691502 (500 nM) inhibited expression of this protein at all time points; p4EBP-1 expression was markedly attenuated at 24 and 48 h and completely inhibited at 72 h. A single treatment with PF-04691502 not only demonstrated sustained inhibition for the 24 h period in QGP-1 and BON cells (Figure 2B), but also attenuated pAkt, pS6 and p4EBP-1 at 24 h in NT-3 cells (data not shown). Moreover, these results demonstrate that PF-04691502 can effectively inhibit PI3K/mTOR pathway components in both QGP-1 and BON cells for at least 72 h.

**Figure 3:**
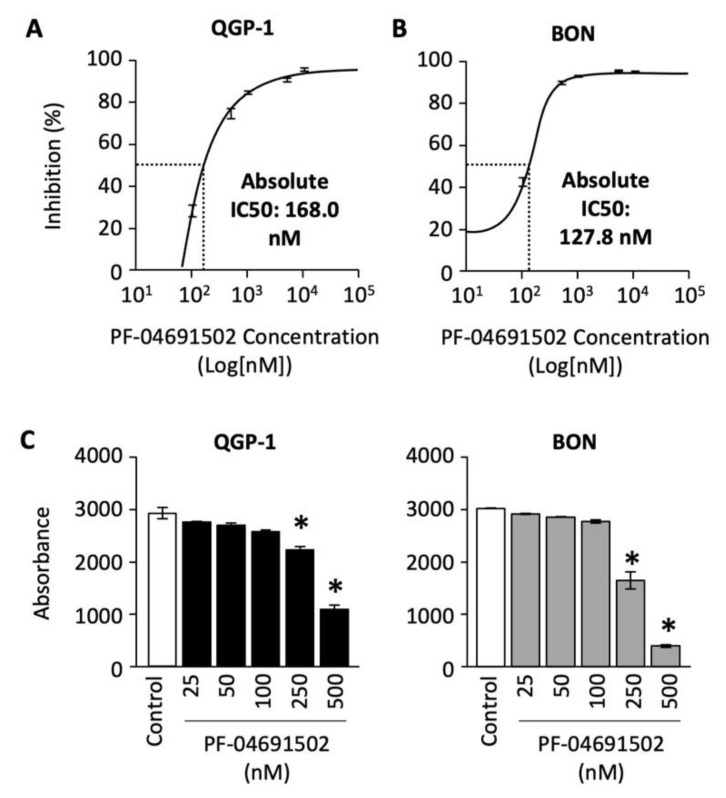
Analysis of QGP-1 and BON cells’ proliferation and cell survival after PF-04691502 treatment. (**A**) QGP-1 and (**B**) BON cells were seeded in 96-well plates at 5000 cells/100 µL density (*n* = 6 per group) and treated with PF-04691502 from 100 to 10,000 nM for 72, 96, and 120 h. Cell proliferation was analyzed with SRB assay as described. Percentage inhibition of proliferation was plotted against concentrations of PF-04691502 on a logarithmic scale. Absolute IC-50 was denoted by a dashed line and labeled, respectively. (**C**) QGP-1 and BON cells were seeded in a 96-well plate at 200 cells/100 µL density (*n* = 6 per group) and treated with PF-04691502 from 25 to 500 nM. Cells were fixed and stained according to SRB protocol 14 d later. *, *p* < 0.01 versus control colorimetric cell survival by one-way ANOVA analysis.

To determine the effect of PF-04691502 on GEP-NET cell proliferation, we treated QGP-1 and BON cells with concentrations ranging from 100 to 10,000 nM for 120 h. The percentage of cell inhibition was plotted against the concentration of PF-04691502 on a logarithmic scale, and a standard curve was fitted to calculate the absolute IC-50 values. A dose-dependent increase in percentage inhibition of cellular proliferation was observed in both QGP-1 and BON cells (Figure 3A,B). The absolute IC-50 value, defined as 50% inhibition compared to control, ranges from 168 nM for QGP-1 cells to 127.8 nM for BON cells.

Next, we evaluated the effect of PF-04691502 on QGP-1 and BON cell survival. Cells were treated with PF-04691502 (from 25 to 500 nM) and cell survival was quantified as previously described. We noted a statistically significant decrease in cell survival at dosages of 250 and 500 nM for both QGP-1 and BON cells (Figure 3C). These results demonstrate significant antiproliferative activity of PF-04691502 in GEP-NET cell lines, particularly at a dosage of 500 nM.

### 3.3. Cellular Profiling of PF-04691502 in Patient-Derived Tumor Spheroid Model

**Figure 4:**
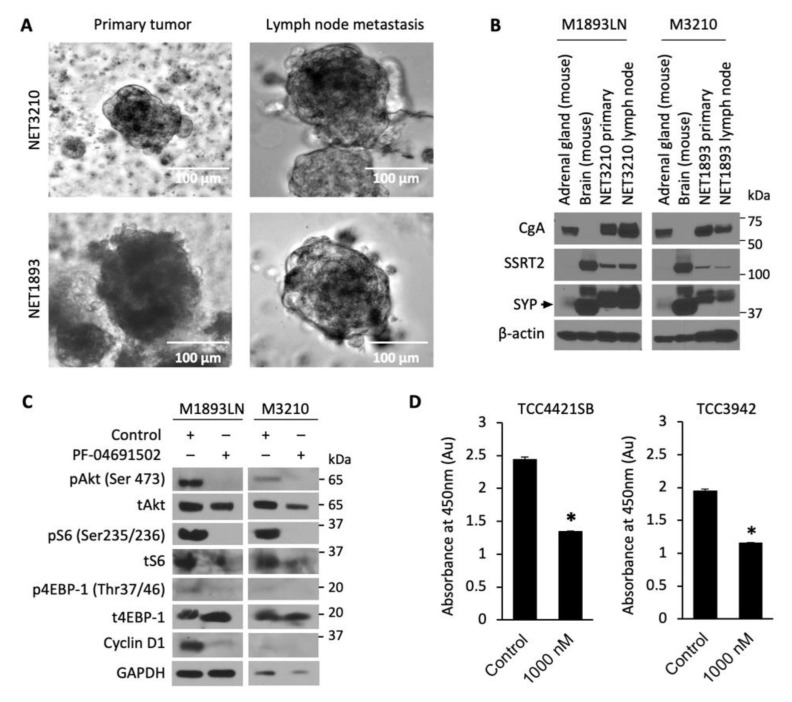
Patient-derived NET tumor spheroids treatment with PF-04691502. (**A**) Photographs of tumor spheroids in low-attachment plates. GEP-NET patient tumor sample M3210 was a pT3N2M1a grade 2 tumor ileal neuroendocrine tumor, and tumor sample M1893 was a pT3N2Mx small intestinal neuroendocrine tumor. (**B**) GEP-NET tumor spheroids were plated in low-attachment plates and 7 d later protein lysates were collected for GEP-NET origin confirmation by Western blot. Mouse brain and mouse adrenal glands were used as positive controls for SYP and CgA, correspondingly. (**C**) GEP-NET tumor spheroids were seeded in low-attachment plates with equal density and treated with 500 nM PF-04691502 for 24 h. Protein lysates were collected to confirm PI3K/Akt pathway inhibition by pAkt (Ser473), pS6 (Ser235/236) and p4EBP-1 (Thr37/46) by immunoblotting. β-actin was used as a loading control. GAPDH was used as a loading control. (**D**) GEP-NET tumor spheroids were seeded in a 96-well clear round-bottom ultra-low attachment microplate at 10,000 cells / 1 mL density and treated 24 h later with 1000 nM PF-04691502. CCK-8 reagent was added to each well 48 h later; CCK-8-induced colorimetric changes were measured by absorbance at 450 nM, which is directly proportional to the rate of cell proliferation, after 72 h. * denotes *p*-value < 0.01.

Developing new preclinical models for GEP-NET is a well-known challenge, as slow growth and genetic stability limit the availability of NET cell lines, PDX and metastatic models [43]. We developed and evaluated 3D patient-derived NET spheroids to perform rapid and reliable evaluation of therapeutics in vitro. GEP-NET tumor samples M1893 primary (pT3N2Mx, well-differentiated small intestinal NET) and M3210 primary (pT3N2M1a, well-differentiated small intestinal NET), with their respective lymph node (LN) metastasis, were established and cultured as tumor spheroids (Figure 4A). The neuroendocrine origin of the tumor spheroids was confirmed by immunoblotting analysis of neuroendocrine biomarkers: chromogranin A (CgA), somatostatin receptor 2 (SSTR2), and synaptophysin (SYP) (Figure 4B). After confirming that these patient-derived spheroids were indeed neuroendocrine in origin, we treated the spheroids with PF-04691502 (500 nM) for 24 h and determined expression of pAkt, pS6, and p4EBP-1. Similar to GEP-NET cell lines, we observed the inhibition of pAkt, pS6, and p4EBP-1 expression in spheroid samples at 24 h. In addition to the inhibition of the PI3K/Akt pathway, we also observed inhibition of cyclin D1 after PF-04691502 treatment for 24 h. The CCK-8 viability assay, performed using two distinct patient-derived spheroid samples, showed a significant 40% decrease in absorbance at 450 nm and thus proportional decrease in proliferation for both patient-derived spheroid samples (Figure 4D). These results demonstrate both the validity of our NET spheroid model as a reproducible tool for the evaluation of novel therapeutics and the anti-proliferative effect of PF-04691502 on GEP-NETs.

### 3.4. Enhanced Radiosensitization of GEP-NET Cells via Schedule Dependent PF-04691502 Treatment

**Figure 5:**
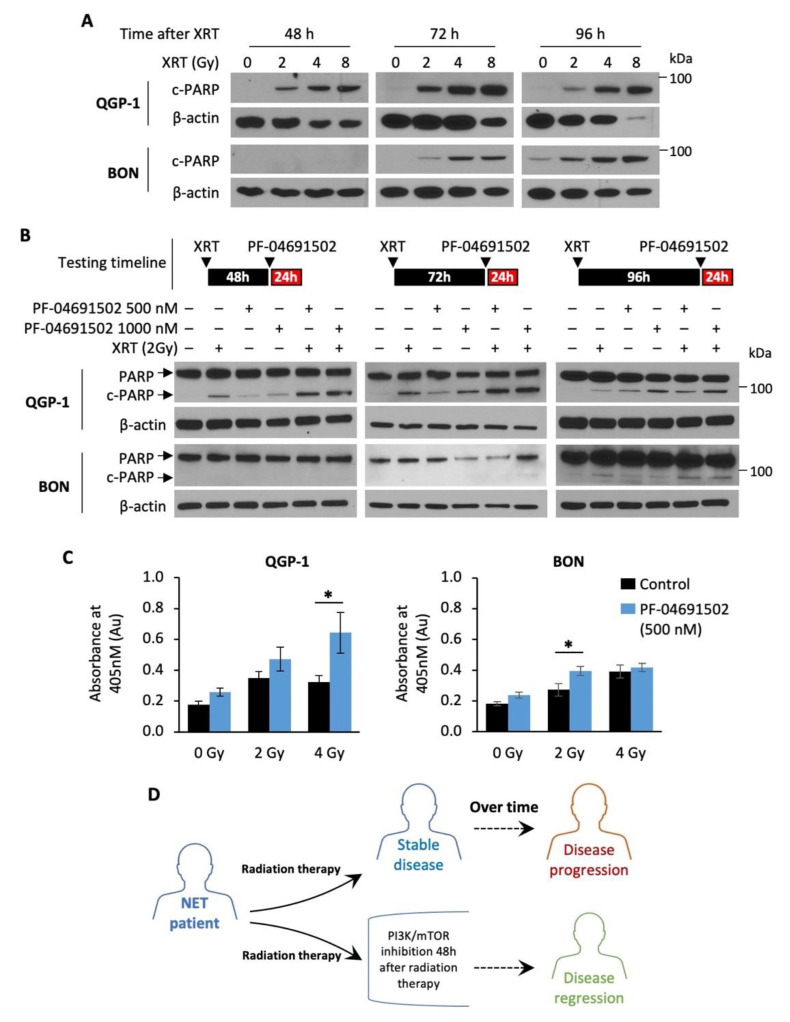
Radiotherapy and schedule-dependent PF-04691502 therapy in NET cells. (**A**) QGP-1 (top) and BON (bottom) cells were seeded in a 6-well plate at 800,000 cells/2 mL density. Cells were then exposed to either control (0 Gy), 2, 4, or 8 Gy X-ray radiation. Protein lysates were collected at 24, 48, 72, and 96 h after radiation exposure and analyzed for cleaved-PARP expression by immunoblotting. β-actin was used as a loading control. (**B**) QGP-1 (top) and BON (bottom) cells were seeded in 6-well plate at 800,000 cells/2 mL density and exposed to 2 Gy X-ray radiation. Cells were incubated for either 48, 72, or 96 h and then treated with PF-04691502 at 500 or 1000 nM for 24 h prior to protein lysates collection. Cleaved-PARP expression was determined by immunoblotting analysis. β-actin was used as a loading control. (**C**) QGP-1 and BON cells were seeded at 1000 cells/100 uL density in 96-well plates and exposed to 2 Gy X-ray radiation. Cells were incubated for 96 h and treated with PF-04691502 at 500 nM for 24 h prior to lysate collection and analysis as described. DNA fragmentation and cell death induced proportional colorimetric change, which was measured by absorbance at 405 nm (*n* = 6). * denotes *p*-value < 0.01. (**D**) Proposed schematic diagram for schedule-dependent PI3K/mTOR inhibition after radiotherapy in NET patients.

Radiotherapy, namely PRRT, has emerged as the next-generation treatment for GEP-NET patients [44,45]. Here, we tested the hypothesis that combination of a PI3K/mTOR inhibitor with radiotherapy may enhance therapeutic effects in GEP-NETs. Radiotherapy alone resulted in a dose-dependent increase in cleaved PARP expression, a marker of apoptosis, in both QGP-1 and BON cells (Figure 5A). Notably, cleaved PARP expression was detected at least 48 h after radiation in QGP-1 cells and 72 h after radiation in BON cells, suggesting delayed radiation-induced apoptosis in GEP-NET cells. Therefore, we hypothesized that NET cells are most sensitive to PI3K/mTOR inhibition after radiotherapy. Next, QGP-1 and BON cells were irradiated (2 Gy) and then treated with PF-04691502 (500 or 1000 nM) for 24 h at 48, 72 or 96 h after radiation. We observed a significant increase in cleaved PARP expression in cells treated with a combination of radiation and PF-04691502 at 48 and 72 h post-radiation in QGP-1 cells and 96 h post radiation in BON cells (Figure 5B). In contrast, increased cleaved PARP expression was not noted in cells treated with 4 Gy radiation, immediately followed by PF-04691502 (500 nM or 1000 nM) treatment for 24 h (Figure S3). Further DNA fragmentation ELISA immunoassay analysis showed increased histone biotin and DNA peroxidase presence, markers of DNA fragmentation and apoptosis, in cells treated with a combination of radiotherapy (2 Gy) and 24 h treatment of PF-04691502 (500 nM) at 96 h after radiation. We observed a statistically significant increase in DNA fragmentation in the 4 and 2 Gy subgroups for QGP-1 and BON cells, respectively (Figure 5C). Our findings suggest that delayed treatment with a PI3K/mTOR inhibitor after radiotherapy can result in increasing cytotoxic effects compared to either drug or radiotherapy alone. Most importantly, our data demonstrate a schedule dependence of apoptosis induction after radiation and PI3K/mTOR inhibition in GEP-NET cells. Therefore, our results suggest the use of a PI3K/mTOR inhibitor in combination with radiotherapy in a delayed fashion as a possible treatment strategy to prevent disease progression and to promote disease regression in NET patients, as proposed in the schematic diagram (Figure 5D).

## 4. Discussion

GEP-NET is a subset of aggressive gastrointestinal NETs, often diagnosed as advanced disease with poor prognosis [3]. Improved treatment options are needed, as a third of GEP-NET patients do not respond to current regimens [2,3,18]. Radiotherapy and mTOR inhibition are two promising therapies that have improved treatment outcomes, such as progression-free survival [4,5,16,19,22]. PRRT is a form of targeted radiotherapy, coupled with either an alpha-emitter or a beta-emitter and a somatostatin analog to induce DNA damage in targeted GEP-NETs overexpressing somatostatin receptors [4,9]. It has become a prevalent treatment among patients with inoperable and metastatic somatostatin receptor-positive GEP-NETs [4,9,46], with clear progression-free survival benefit and improved quality of life among patients [47,48]. Despite the fact that the initial PRRT treatment resulted in stable disease in up to 80% of patients, all patients eventually progressed over time [4,5,9,47,49].

Our study explored the therapeutic potential of inhibiting the PI3K/mTOR/Akt pathway, which plays a crucial role in NET pathogenesis [14,49]. The mTOR pathway, one of the most significant targets in modern cancer treatment [14,50], is triggered by growth factors and is responsible for cell survival, proliferation and angiogenesis [14]. There are multiple downstream targets of the mTOR pathway, such as the ribosomal protein S6 kinase 1 (S6K1) and eukaryotic translation initiation factor 4E (eIF4E)-binding protein 1 (4E-BP1) [51]. The S6 protein is a key regulator of 40S ribosome subunit biogenesis and 4E-BP1 has a critical role in translational mRNA complex assembly [50,52,53]. Together, these two proteins are phosphorylated by mTORC1 to promote translation and protein synthesis [50,53]. Clinically, inhibition of S6 and 4E-BP1 phosphorylation are two important therapeutic targets for multiple malignancies [51,52,54,55]. Everolimus, a rapamycin analog and potent mTORC1 inhibitor [56], was used to treat GEP-NETs in the RADIANT clinical trial series [15,16,17,18,19,20]. Though everolimus showed improved progression-free survival in the short term, only 10% of patients had disease regression [15]. This could be secondary to the negative feedback loop of the mTORC2/Akt pathway [57]. The protein S6K inhibits the mTORC2 complex and Akt, which forms the negative feedback loop. Therefore, inhibition of S6K by mTORC1 inhibitor could result in Akt pathway activation through mTORC2 signaling, thus providing an escape mechanism and incomplete inhibition of downstream effectors [57]. Therefore, a new class of PI3K/mTOR dual inhibitors was developed to provide complete inhibition of the mTOR/Akt pathway [24,54].

In contrast to everolimus, the PI3K/mTOR dual inhibitors, PF-04691503 and PKI-402, tested in this study, primarily inhibit PI3K and Akt phosphorylation, an upstream mediator of both mTORC1 and mTORC2 complexes [24]. Inhibition of Akt results in dual inhibition of the mTORC1 and mTORC2, thus preventing the escape negative feedback loop [54]. Our study identified abundant expression of pAkt in the cytoplasm of GEP-NET, which is the primary target of the PI3K/mTOR dual inhibitor (see Figure 1). We also demonstrated significant and prolonged attenuation of pAkt, pS6, and p4EBP-1 expression and antiproliferative properties of the PI3K/mTOR dual inhibitors in vitro. This is consistent with previous studies where PI3K/mTOR dual inhibitors showed antineoplastic properties in various cancer cell lines [58,59,60,61,62]. Despite the abundant supporting evidence for their antineoplastic properties, multiple clinical trials of PI3K/mTOR dual inhibitors reported unacceptable toxicity, resulting in the early termination of trials, including one phase II study in pNET [26,28,63,64]. Thus, it has a limited role in cancer treatment as a single agent [24,25,58]. However, its role as a radiosensitizer has not been previously studied.

Radiotherapy, which utilizes ionized radiation, directly or indirectly damages cellular DNA in cells undergoing rapid division and activates cell survival pathways [65,66]. The ionizing energy can break the double helix DNA structure, which subsequently results in programmed cell death if not repaired successfully, or induce single strand DNA break, which leads to prolonged cell autophagy or cell cycle arrest [66]. When cells sustain DNA damage, the non-homologous end joining (NHEJ) or homologous recombination (HR) repair pathways become activated and phosphorylate ATR, ATM and DNA-PK to promote cell survival [65,66]. The PI3K/mTOR/Akt pathway responds to ATM and DNA-PK phosphorylation through AMPK signaling, and triggers its downstream effectors to promote survival, proliferation and angiogenesis, as described previously [65]. This forms the basis for our hypothesis that PI3K/mTOR dual inhibitor could enhance the cytotoxic effect of radiotherapy.

Our laboratory recently showed that Akt1 expression plays a major role in the radiosensitivity of triple negative breast cancer [38]. Therefore, we hypothesized that combining radiotherapy and PI3K/mTOR dual inhibitor could prevent the cell survival response to DNA damage from radiation therapy, thus achieving synergistic cytotoxic effect through a multimodal approach. We used PI3K/mTOR dual inhibitors, which prevent re-activation of mTOR through negative feedback, and combined it with radiotherapy to induce synergistic apoptosis in GEP-NET cell lines. In addition to the radiosensitization by PI3K/mTOR inhibitions, our study identified a schedule-dependent induction of apoptosis. Pre-radiation and simultaneous treatment with PF-04691502 did not increase apoptosis, while PF-04691502 administered post-radiation produced a synergistic induction of apoptosis compared to either PF-4691502 or radiation alone. Moreover, cells did not undergo apoptosis immediately after radiation. In contrast, there was a delayed apoptotic response at 48 h and at longer time points. Prior preclinical studies have also noted a schedule-dependent radiosensitization using sorafenib and erlotinib [67,68]. One study suggested that this variation could be due to PI3K signal transduction and its regulatory effects [68]. Schedule-dependent PI3K/mTOR inhibition has a potential to reduce the frequency of side effects observed after PI3K/mTOR pathway inhibition by reducing number of administered doses required to achieve cytotoxic effects in cancer cells.

In summary, we demonstrate that PF-06491502 is a long-acting PI3K/mTOR inhibitor with antineoplastic activity, which can also potentiate radiotherapy when administered in a schedule-dependent fashion. Our findings suggest that PI3K/mTOR inhibitors should be administered in the short term after radiation therapy to potentiate radiotherapy and to possibly improve clinical outcomes in GEP-NET patients. Short-term exposure of PI3K/mTOR inhibitor administration in this setting could prevent toxicity of inhibitors associated with long-term treatment.

## Data Availability

Research data are stored in an institutional repository and will be shared upon request to the corresponding author.

## Supplementary Materials

The following are available online at https://www.mdpi.com/article/10.3390/cells10051261/s1, Figure S1: pAkt (Ser473) expression pNET patient tumor samples, Figure S2: pAkt (Ser473) inhibition by PKI-402 in NET cell lines at 24 h, Figure S3: Simultaneous PF-04691502 therapy and radiation therapy in NET cell lines.
