# Supplementary material for: PI3K/mTOR Dual Inhibitor PF-04691502 Is a Schedule-Dependent Radiosensitizer for Gastroenteropancreatic Neuroendocrine Tumors
Source: Cells. 2021 May 20;10(5):1261. doi: 10.3390/cells10051261 (PMC8160730; doi:10.3390/cells10051261)
Supplement: Supplementary file 1 [file cells-10-01261-s001.zip › cells-1141310-supplementary.pdf]

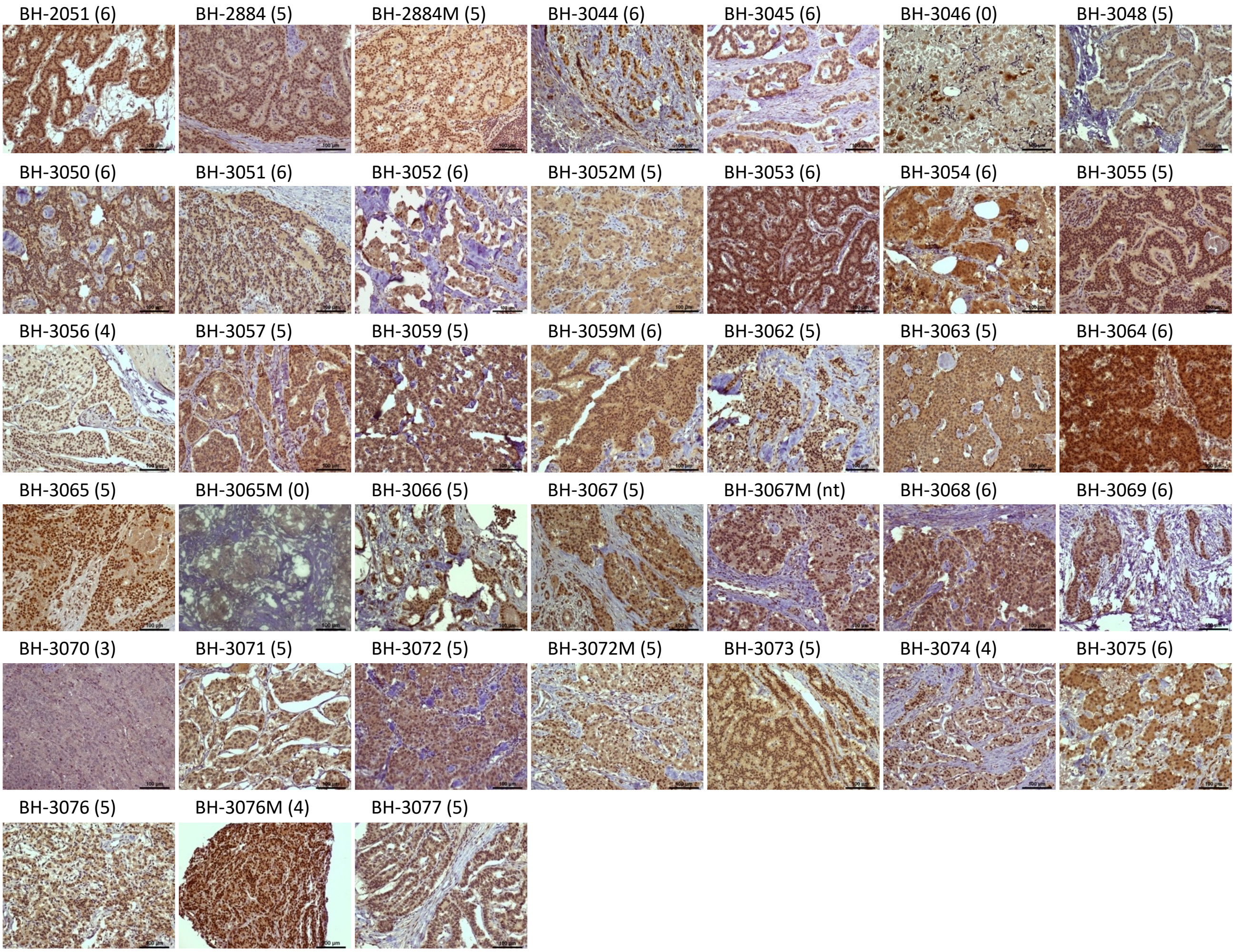

Figure S1. pAkt (Ser473) expression pNET patient tumor samples. IHC staining of pAkt (Ser473) in pNET primary and metastatic tumor samples. Samples were scored on scales of 0-3 for both intensity and distribution percentage and then added together (n = 39). Total IHC staining scores are shown in parentheses (nt indicates no tumor).

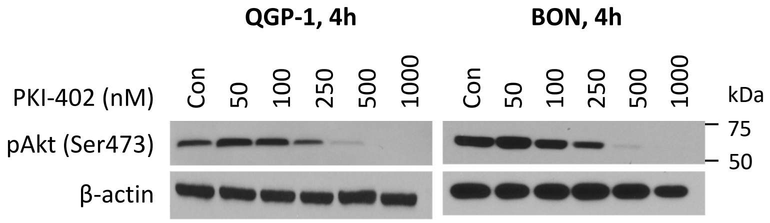

Figure S2. pAkt (Ser473) inhibition by PKI-402 in NET cell lines at 24h. (A) QGP-1 (left) and BON (right) cells were seeded in a 6-well plate at 800,000 cells per well density and treated with PKI-402 from 50nM to 1,000nM, and collected at 4h. Immunoblotting was performed to confirm PI3K/Akt pathway inhibition at 4h. β-actin was used as a loading control.

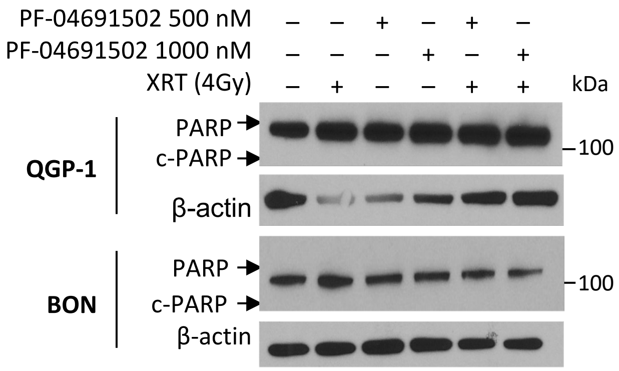

Figure S3. Simultaneous PF-04691502 therapy and radiation therapy in NET cell lines. QGP-1 (top) and BON (bottom) cells were seeded in 6-well plate at 800,000 cells per well density and exposed to 4 Gy X-ray radiation. Cells were then immediately treated with PF-04691502 at 500nM or 1,000nM for 24h prior to protein collection. Cleaved-PARP expression was determined by immunoblotting. β-actin was used as a loading control.
